# A Closed-Loop Toolchain for Neural Network Simulations of Learning Autonomous Agents

**DOI:** 10.3389/fncom.2019.00046

**Published:** 2019-08-02

**Authors:** Jakob Jordan, Philipp Weidel, Abigail Morrison

**Affiliations:** ^1^Department of Physiology, University of Bern, Bern, Switzerland; ^2^Institute of Neuroscience and Medicine (INM-6) & Institute for Advanced Simulation (IAS-6) & JARA-Institute Brain Structure Function Relationship (JBI 1/INM-10), Research Centre Jülich, Jülich, Germany; ^3^aiCTX, Zurich, Switzerland; ^4^Department of Computer Science, RWTH Aachen University, Aachen, Germany; ^5^Faculty of Psychology, Institute of Cognitive Neuroscience, Ruhr-University Bochum, Bochum, Germany

**Keywords:** closed-loop simulation, reinforcement learning, spiking neuronal networks, virtual environments, computational neuroscience

## Abstract

Neural network simulation is an important tool for generating and evaluating hypotheses on the structure, dynamics, and function of neural circuits. For scientific questions addressing organisms operating autonomously in their environments, in particular where learning is involved, it is crucial to be able to operate such simulations in a closed-loop fashion. In such a set-up, the neural agent continuously receives sensory stimuli from the environment and provides motor signals that manipulate the environment or move the agent within it. So far, most studies requiring such functionality have been conducted with custom simulation scripts and manually implemented tasks. This makes it difficult for other researchers to reproduce and build upon previous work and nearly impossible to compare the performance of different learning architectures. In this work, we present a novel approach to solve this problem, connecting benchmark tools from the field of machine learning and state-of-the-art neural network simulators from computational neuroscience. The resulting toolchain enables researchers in both fields to make use of well-tested high-performance simulation software supporting biologically plausible neuron, synapse and network models and allows them to evaluate and compare their approach on the basis of standardized environments with various levels of complexity. We demonstrate the functionality of the toolchain by implementing a neuronal actor-critic architecture for reinforcement learning in the NEST simulator and successfully training it on two different environments from the OpenAI Gym. We compare its performance to a previously suggested neural network model of reinforcement learning in the basal ganglia and a generic Q-learning algorithm.

## 1. Introduction

Simulation is a key component of modern neuroscience, constituting a third methodological pillar along with experiment and theory. Its uses include, but are not limited to, validation of theory, generation of hypotheses, production of surrogate data for data analysis tools, and discovery of structural and dynamical constraints for functional models. Thanks to a variety of initiatives, researchers now have access to well maintained, high performance simulators for all scales of neural systems from molecular simulations (e.g., STEPS; Wils and De Schutter, 2009) over complex neuron (e.g., NEURON, Carnevale and Hines, 2006; GENESIS, Bower and Beeman, 2007) and network models (e.g., NEST, Gewaltig and Diesmann, 2007; BRIAN, Goodman and Brette, 2009; NENGO, Bekolay et al., 2013, SINABS, Sheik and Liu, 2019) to whole brain simulations using neural fields (e.g., TVB, Sanz Leon et al., 2013).

In the realm of spiking neural networks, development of simulators has been largely driven by two viewpoints: the physical, concerned with the dynamics of individual neurons and networks of neurons (e.g. relationship of correlation structure to connectivity, bifurcation landscapes), and the electrophysiological, concerned with the response of neurons and networks to stimuli (e.g. PSTHs, response variability). Thus, spiking neural network simulators provide good support for constructing structured networks of neurons with a variety of dynamics, applying arbitrarily complex stimuli and recording the evolution of dynamic variables for later analysis.

However, restricting our inquiries to the dynamical and transformational properties of neuronal networks neglects large classes of fundamental and exciting neuroscientific questions. In particular, investigations of embodied cognition, of organisms operating autonomously in an environment and learning how to optimize their behavior within it, require a different approach. Firstly, it is crucial to simulate agents that interact with their environments, thereby actively shaping their future sensations rather than merely passively consuming experimentally provided stimuli (see e.g., Wilson, 2002). This necessitates a *closed-loop* set-up, in which the neuronal network can be conceived of as an autonomous agent within an environment. The neuronal network receives sensory stimuli from the environment, which alter its network dynamics. The resulting activity of the network, or of specific subnetworks of it can be interpreted as motor commands which alter the agent's configuration with respect to its environment (e.g., rotation, lateral movement) or the configuration of the environment itself (e.g., operation of levers or buttons). The change in configuration brings about a change in the sensory stimuli, and thus the neuronal network interacts with the environment in a continuous cycle. Depending on the scientific question, the network activity can also drive plasticity processes in the network, causing alterations in its own configuration, and thus in its response to sensory stimuli. In this way, new behavior can be learned through interaction with the environment, rather than through extensive exposure to labeled training data.

Secondly, it is important to establish a set of standardized benchmarks which allow alternative models to be compared with each other and good models to be improved and extended. With regard to this latter point, a comparison of the progress of the fields of machine learning, and learning in neuronal networks, provides a useful illustration. The last decade has witnessed major progress in the field of machine learning, moving from small-scale toy problems to large-scale real-world applications including image (Krizhevsky et al., 2012) and speech recognition (Hinton G. et al., 2012), complex motor-control tasks (Mnih et al., 2016), and playing (video) games at super-human performance (Mnih et al., 2015; Silver et al., 2016). This progress has been driven mainly by an increase in computing power, especially by training deep networks on graphics processing units (Raina et al., 2009), and conceptual breakthroughs like layer-wise pretraining (Hinton and Salakhutdinov, 2006; Bengio et al., 2007) or dropout (Hinton G.E. et al., 2012). Even so, this rate of progress would not have been possible without the wide availability of high-performance ready-to-use tools, e.g., Torch (Collobert et al., 2002), Theano (James et al., 2010), Caffe (Jia et al., 2014), TensorFlow (Abadi et al., 2016), and standardized datasets and environments for benchmarking, such as the MNIST (LeCun et al., 1998), CIFAR (Krizhevsky and Hinton, 2009), and ImageNET (Deng et al., 2009) datasets, and the MuJoCo (Todorov et al., 2012), ALE (Bellemare et al., 2015), and OpenAI Gym (Brockman et al., 2016) toolkits. While ready-to-use tools allow researchers to focus on important aspects rather than basic implementation details, standardized benchmarks have guided the community as a whole toward promising approaches, as for example in the case of convolutional networks through the ImageNET competition (Russakovsky et al., 2015).

Similarly, researchers in the field of computational neuroscience have benefited from the increase of computational power and achieved many conceptual breakthroughs over the last decade, with a plethora of new neuron, synapse and network models being developed. As mentioned above, a variety of simulators are available to the computational neuroscientist, yet so far no generally accepted set of benchmarks exist (but see Gerstner and Naud, 2009).

One particular area in which the lack of standardized benchmarks is apparent is research into reinforcement learning (RL) in neurobiological substrates. Inspired by behavioral experiments, RL is concerned with the ability of organisms to learn from previous experiences to optimize their behavior in order to maximize reward and avoid punishment (see e.g., Sutton and Barto, 1998). RL has a long tradition in the field of machine learning which has led to several powerful algorithms, such as SARSA and Q-learning (Watkins, 1989). Similarly, a large variety of neurobiological models have been proposed in recent years (Izhikevich, 2007; Potjans et al., 2009, 2011; Urbanczik and Senn, 2009; Vasilaki et al., 2009; Frémaux et al., 2010; Frémaux et al., 2013; Jitsev et al., 2012; Friedrich et al., 2014; Rasmussen and Eliasmith, 2014; Aswolinskiy and Pipa, 2015; Baladron and Hamker, 2015; Rombouts et al., 2015; Friedrich and Lengyel, 2016; Rueckert et al., 2016). However, only a small proportion of these rely on publicly available simulators and all of them employ custom built environments. Even for fairly simple environments, this has led to a situation where different network models are difficult to compare and reproduce, thus creating a fragmentation of research efforts. Instead of building upon and extending existing models, researchers are forced to spend too much time on recreating basic functionality for custom implementations.

The need for closed-loop simulation has led to the Human Brain Project (2014) (HBP) dedicating significant resources of a subproject (Neurorobotics) to the development of the necessary infrastructure that allows users to conduct robotic experiments in virtual environments and connect these to their neural network implementations with a web interface (Falotico et al., 2017). This approach specifically addresses the need of researchers developing neuronal or neuro-inspired controllers for robotic applications. A more pared-down approach, suitable for researchers who are primarily concerned with understanding the neural circuits, rather than controlling sophisticated robotic actuators, is provided by Weidel et al. (2016). This approach allows any neuronal network simulator that implements the MUSIC (Djurfeldt et al., 2010) interface (including NEST and NEURON) to be coupled with any robotic simulator implementing the ROS (Quigley et al., 2009) interface [including Gazebo (Koenig and Howard, 2004), Morse (Echeverria et al., 2011), or Webots (Michel, 2004)].

However, neither approach directly addresses the issue of the lack of standardized benchmarks for neuronal agents operating autonomously and learning to optimize their behavior in an environment. Such benchmarks exist: the OpenAI Gym (Brockman et al., 2016) provides a rich and generic collection of standardized RL environments developed to support the machine learning community in evaluating and comparing algorithms. All environments are accessible via a simple, unified interface, that requires an agent to supply an action and returns an observation and reward for its current state. The toolkit includes a range of different environments with varying levels of complexity ranging from low-dimensional fully discrete (e.g., *FrozenLake*^1^) to high-dimensional fully continuous tasks (e.g., *Humanoid*^1^). The consistency of the OpenAI Gym environments across different releases supports researchers in reproducing and extending previous work and allows systematic benchmarking and comparison of learning algorithms and their implementations. The easy accessibility of different tasks fosters progress by allowing researchers to focus on learning algorithms instead of basic implementation details of particular environments, and prompts researchers to evaluate the performance of their algorithms on many different tasks.

One possibility to access this set of benchmarks is to implement spiking networks in tools that are natively compatible with the OpenAI Gym, such as Tensorflow (Abadi et al., 2016) or PyTorch (Paszke et al., 2017). However, as the components of spiking neural network models (e.g., neuron and plastic synapse models, stimulation, and recording devices) are typically not shipped with these tools, this once again places the burden of implementation on the user (but see Hazan et al., 2018 for a spiking-neural network orientated approach). In particular, since these tools focus on machine learning applications rather than exploring biological intelligence, several critical features for computational modeling of learning in biological neuronal networks, such as few-compartment neurons, conductance-based synaptic interactions or neuromodulated plasticity, lie outside the scope of these libraries. Therefore, to make a comprehensive resource of benchmarks available to the computational neuroscience community, we developed a toolchain to interface neural network simulators with the OpenAI Gym. Using this toolchain, researchers can rely on well-tested, high-performance simulation engines for spiking neural networks to power their models, and evaluate them against a curated set of standardized environments, allowing more time to focus on neurobiological questions, such as the configuration and plasticity of neural circuits underlying exploration of the environment and exploitation of prior experience.

In the next section we introduce additional pre-existing components on which our toolchain relies, and afterwards discuss how it links the different tools. We demonstrate its functionality by implementing a neural actor-critic in NEST and successfully training it on two different environments from the OpenAI Gym.

## 2. Pre-existing Components

All network simulations in this manuscript are carried out with NEST^2^ (Gewaltig and Diesmann, 2007), a neural simulator designed for the efficient simulation of large-scale networks of simple spiking neuron models with biophysically realistic connectivity. The simulation kernel scales from small simulations on a laptop to super computers, with the largest simulation to date containing about 10^9^ neurons and 10^13^ synapses, corresponding to about 10% of the human cortex at the resolution of individual cells and connections (Kunkel et al., 2014; Jordan et al., 2018). NEST is actively developed and maintained by the NEST initiative^3^ in collaboration with the community, is freely available under the GPLv2 and is supported by the HBP with the explicit aim of widespread long-term availability and maintainability. The simulation set-up, e.g., definition of neurons and connections, can conveniently be performed via an interpreted language (e.g., PyNEST; Eppler et al., 2009) while the propagation of network dynamics is implemented in C++. OpenMP is used for node-local parallelization while MPI provides inter-node communication. While using a compiled language for the compute-intensive part provides significant performance gains compared to an interpreted language, it makes it less straightforward to interface the simulator with other tools not specifically designed for this.

The OpenAI Gym (Brockman et al., 2016) is a toolkit for reinforcement learning research focused on ease of use for machine learning researchers. An explicit goal of the OpenAI Gym is to compare different RL algorithms with each other in a consistent fashion. It provides a unified Python interface to a rich collection of curated RL environments, e.g., Atari games^4^ or continuous control tasks for robotic applications^5^.

An environment in the OpenAI Gym is updated in steps. In each step, the agent receives an observation representing the state of the environment, e.g., the agent's location within it, or other configurational information. This is typically a vector of real values. In addition, it receives a real-valued reward for entering the current environmental state. Depending on the environmental set-up, the reward may be zero for the majority of state transitions, and only non-zero (positive for rewards or negative for punishments) when the agent achieves a well-defined goal. On the basis of the current state and its internal policy, the agent provides an action to the environment to trigger the next state transition. The reward can be used as information to adjust the agent's policy, such that its behavior in the environment evolves, typically such that it receives more reward in future trials in the same environment.

While the network implementation that we present in the results section relies on the NEST simulator, the toolchain can also be used with other simulators that support the MUSIC library, for example NEURON (Carnevale and Hines, 2006). The MUlti-SImulation Coordinator is a multi-purpose middleware for neural network simulators built on top of MPI (Message Passing Interface) that enables online interaction of different simulation engines (Djurfeldt et al., 2010). MUSIC takes care of starting all MUSIC-controlled executables (e.g., adapters and simulators) defined in a configuration file provided by the user in separate processes. During execution it makes sure that all processes evolve synchronously with a predefined real-time factor independent of the computational load of the individual processes (Moren et al., 2015). MUSIC provides named MPI channels, referred to as MUSIC ports, which allow the user to set up communication streams between several processes. While originally intented to distribute a single neural network model across different simulators, the MUSIC library can also be used to connect neural simulators to other applications.

For example, to connect neural simulators to robotic simulators, we recently developed the ROS-MUSIC Toolchain (RMT; Weidel et al., 2016) which provides an interface from MUSIC to the Robotic Operating System (ROS; Quigley et al., 2009). ROS is the most popular middleware in the robotic community and is able to interact with many robotic simulators and hardware platforms. The RMT allows exchange of well-defined messages between ROS and MUSIC via stand-alone executables, so called adapters, that were designed with a focus on modularity. The toolchain contains several different adapters each performing a rather simple operation on streams of inputs (e.g., filtering). By concatenating several adapters, the overall transformation of the original data can become more complex, for example converting high-dimensional continuous data (e.g., sensory data) to low-dimensional discrete data (e.g., action potentials) or vice-versa. More information and introductory examples can be found on GitHub^6^.

## 3. Results

To enable the online interaction of neural network simulators and the OpenAI Gym, we rely on two different libraries: MUSIC, to interface with the neural simulator, and ZeroMQ (Hintjens, 2013) to exchange messages with the environment simulated in the OpenAI Gym. In the following, we describe these two parts of the toolchain and demonstrate their functionality by interfacing a neural network simulation in NEST with two different environments.

### 3.1. Extending the ROS—MUSIC Toolchain

We extended the RMT by adding adapters that support communication via ZeroMQ following a publish-subscribe pattern. ZeroMQ is a messaging library that allows applications to exchange messages at runtime via sockets. Continuously developed by a large community, it offers bindings for a variety of languages including C++ and Python, and supports most operating systems. A single communication adapter of the RMT sends (receives) data via a ZeroMQ socket and receives (sends) data via a MUSIC port. While the adapters can handle arbitrary data, we defined a set of specialized messages in JSON format (see Supplementary Material) specifically designed to communicate observations, rewards, and actions as discrete or continuous real-valued variables of arbitrary dimensions, as used in the OpenAI Gym. We chose the JSON format due to its simplicity, easy serialization and broad platform support.

In addition to the ZeroMQ adapters dedicated for communication with MUSIC, we developed several further adapters that can perform specific transformations of the data. OpenAI Gym places few restrictions on the nature of the environment: it can be continuous or discrete with arbitrary dimensionality. Thus, in order to generate the required closed-loop functionality, the observations provided by the environment must be consistently transformed to a format that can be fed into neural network simulations. Conversely, the activity of the neural network must be interpreted and transformed into valid actions which can be executed in the environment.

A standard way to address the first issue with some degree of biological plausibility is to introduce a layer of *place cells* (Moser et al., 2008). Each of these cells is tuned to a preferred (multidimensional) observation, i.e., is highly active for a specific input and less active for other inputs (see e.g., Frémaux et al., 2013). The dependence of the activity of a single place cell on observations is described by its tuning curve, often chosen as a multidimensional Gaussian. To perform the transformation of observations to activity of place cells, we implemented a *discretize adapter* that allows users to specify the position and width of the tuning curves of an arbitrary number of place cells. One disadvantage of this approach is that the number of place cells required to cover the whole observation space evenly scales exponentially in the number of dimensions of the observation. For observations with a small number of dimensions, however, this approach is very suitable.

To perform action selection, we added several adapters that can, respectively, select the most active neuron (*argmax adapter*), threshold the activity across neurons to create a binary vector (*threshold adapter*), or linearly combine the activity of neurons across many input channels (*linear decoder*). Depending on the type of action required by the environment (discrete/continuous), the user can select a single one or a combination of these. Specifications of the adapters can be found in the documentation of the RMT^7^.

In general, we followed the design principle behind the RMT and developed modular adapters. This makes each individual adapter easy to understand and enables users to quickly extend the toolchain with their own adapters. By combining several adapters, the RMT allows arbitrarily complex transformations of the data and can hence be applied to many use-cases.

### 3.2. ZeroMQ Wrapper for the OpenAI Gym

**Figure 1:**
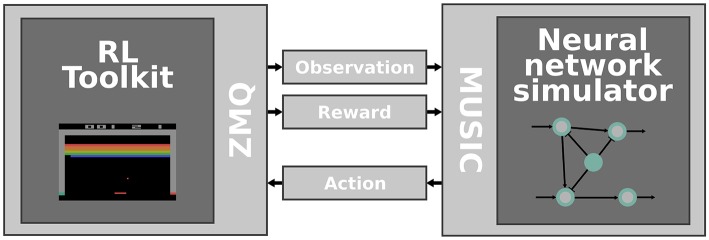
Interfacing RL toolkits with neural network simulators. The RL toolkit **(left)** is responsible for emulating an environment that provides observations and rewards which are communicated via ZeroMQ sockets and MUSIC adapters **(middle)** to a neural network simulator **(right)**. Concurrently, the activity of the simulated neural network is transformed to an action and fed back to the RL toolkit.

The second part of the toolchain is a Python wrapper around the OpenAI Gym that exposes ZeroMQ sockets (Hintjens, 2013) for communicating actions, observations and rewards (see section 2 and Figure 1). The wrapper consists of four different threads that coordinate: (i) performing steps in an environment, (ii) receiving actions via a ZeroMQ SUB socket, (iii) publishing observations via a ZeroMQ PUB socket, and (iv) publishing rewards via a ZeroMQ PUB socket.

Before spawning the threads, the wrapper starts a user-specified environment and creates the necessary communication buffers. The thread coordinating the environment reads actions from the corresponding buffer, performs single steps in the environment and updates the observation and reward buffers based on the return values of the environment. Upon detecting that a single episode has ended, e.g., by an agent reaching a certain goal position, it resets the environment and allows a break of user-specified duration before starting the next episode.

The communication threads continuously send (receive) messages via ZeroMQ and read from/write to the corresponding buffers. All threads can be run with different update intervals, for example, to slow down movement of the agent by performing steps on a coarse time grid whilst continuously receiving action choices from the neural network simulation running on a fine time grid. The user can specify a variety of parameters via a configuration file in JSON format (see Supplementary Material). Detailed specifications of the wrapper can be found in the documentation.

In contrast to MUSIC-controlled executables, the ZeroQM wrapper is not started by the MUSIC library. As a result, the environment and the simulation evolve simultaneously but asynchronously. The simulator hence continuously receives input from the environment and vice versa. Due to the possibility of choosing a real-time factor for MUSIC-controlled processes, the user can easily achieve reliable interaction between the environments and the network simulation. The loosely coupled, asynchronous nature of the toolchain has the benefit that one could, for example, train the same network on a wide variety of different environments without stopping the simulation, in order to investigate transfer learning in spiking neural networks.

### 3.3. Applications

To demonstrate the functionality of the toolchain, we implemented a neural network in NEST and trained it on two different environments simulated in the OpenAI Gym. In the first task the agent needs to learn to perform a sequence of actions in order to reach the top of a hill in a continuous environment. The second task is a classical grid-world in which an agent needs to learn to navigate to a goal position in a two-dimensional discrete environment with obstacles. We first describe the neural network architecture and learning rule and afterwards discuss the network's performance on the two tasks.

#### 3.3.1. Neural Network Implementation

We consider a temporal-difference learning algorithm (Sutton and Barto, 1998) implemented as an actor-critic architecture based on the spiking neuronal network proposed by Frémaux et al. (2013). For the purpose of demonstrating the toolchain, we simplified the model by replacing the spiking neuron models with rate neurons, thereby avoiding issues arising from noise introduced by spiking neuron models (Potjans et al., 2011; Frémaux et al., 2013). Note, however, that the toolchain is not restricted to rate-based models; any neuron model available in the neural simulators with MUSIC interfaces can be used.

The neuron dynamics we considered here are given by the following stochastic differential equation:

(1) $$\begin{array}{l} \tau \frac{dz_{i}\left(t\right)}{dt}=-z_{i}\left(t\right)+\mu_{i}+f\left(h_{i}\left(t\right)-\theta_{i}\right)+\xi_{i}\left(t\right), \end{array}$$

where τ is some positive time constant, μ_*i*_ a baseline activity level, *f*(·) some (arbitrary) activation function, *h*_*i*_(*t*) a time dependent input field, θ_*i*_ an input threshold and ξ_*i*_(*t*) Gaussian white noise with a certain standard deviation σ_ξ_. The input field *h*_*i*_(*t*) is determined by the activity of other neurons according to $h_{i}\left(t\right):=\underset{j}{\sum }w_{ij}z_{j}\left(t\right)$, with *w*_*ij*_ denoting the strength of the connection (weight) from neuron *j* to neuron *i*. Here we will exclusively consider activation functions of the form *f*(*x*): = *x* (linear case), and *f*(*x*): = Θ(*x*)*x* (threshold-linear case, “relu”). Here Θ(·) denotes the Heaviside function, defined as

(2) $$\begin{array}{l} \Theta (x):=\{\begin{array}{l} 1\;x>0\\ 0\text{ else} \end{array} \end{array}$$

Neuron dynamics are integrated in NEST on a fixed time-grid by a stochastic-exponential-Euler method with a step size determined by the resolution of the simulation. For more details on the neuron model implementation (see Hahne et al., 2017).

**Figure 2:**
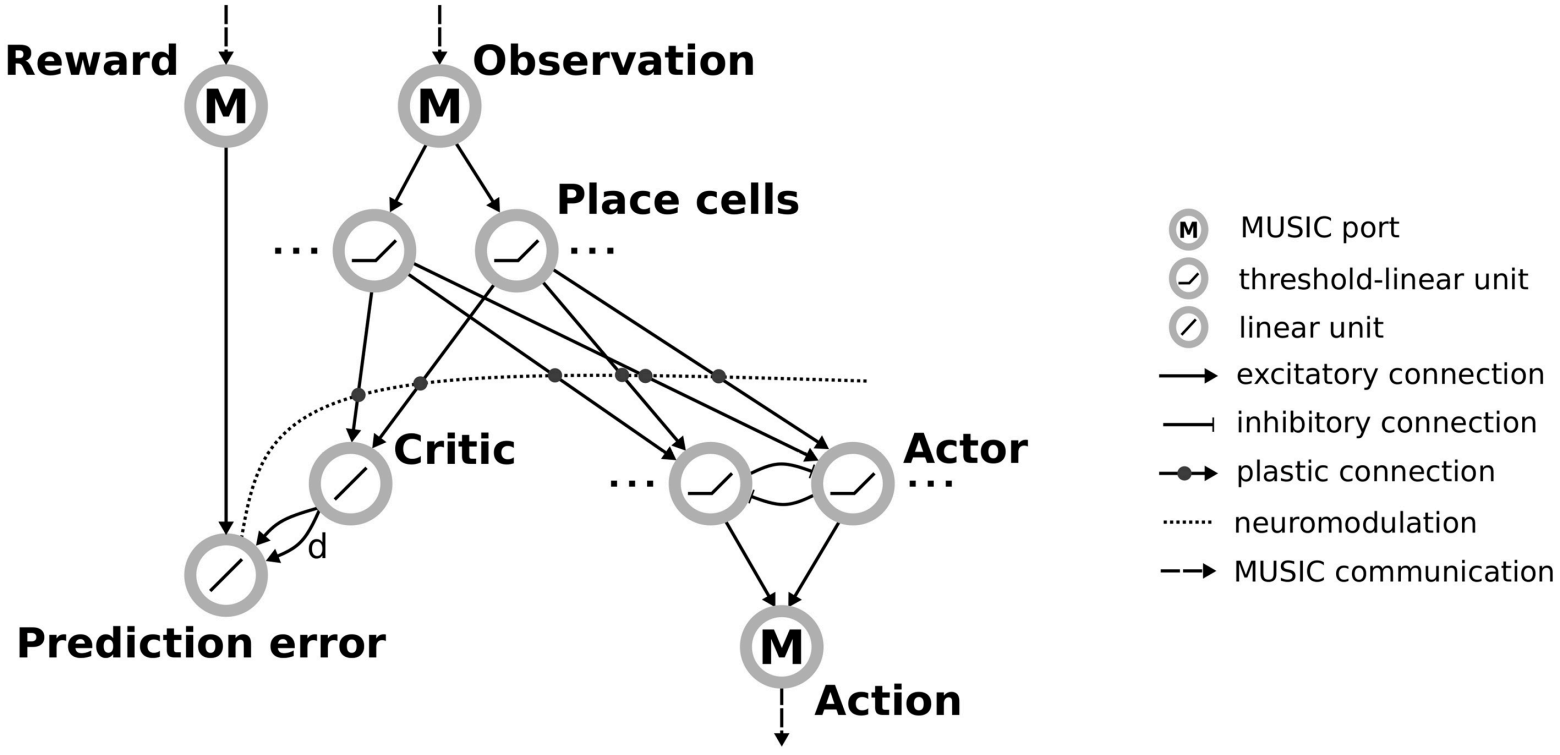
Actor-critic architecture for reinforcement learning with rate neurons. Observations are communicated via a MUSIC input port to a population of place cells. These project both to a critic unit and to actor units arranged in a winner-take-all circuit. The critic and an additional MUSIC input port project to a unit representing the reward prediction error that modulates the plasticity between the place cells and their downstream targets, the critic and actors. The actor units project to a MUSIC output port encoding the selected action.

The input layer is a population of threshold-linear rate neurons which receive inputs through MUSIC and encode observations from the environment (see Figure 2). These place cells project via plastic connections to a single neuron representing the value that the network assigns to the current state (the *critic*). An additional neuron calculates the reward-prediction error by combining the reward received from the environment with input from the critic. Plasticity of the projections from inputs to the critic is modulated by this reward prediction error, as described below.

In addition, neurons in the input layer project to a population of neurons representing the available actions (the *actor*). To enforce selection of a specific action, the actor units are arranged in a winner-take-all (WTA) circuit. This is implemented by recurrent connections between actor units that correspond to short-range excitation and long-range inhibition, the distance reflecting the similarity of the action that actor units encode. The activity of actor units is transformed to an action supported by the environment and communicated to the environment via the RMT.

To derive a learning rule for the critic, we follow similar steps as described by Frémaux et al. (2013), but applied to rate models (Equation 1). The critic activity should approximate a continuous-time value function defined by Doya (2000):

(3) $$\begin{array}{l} V^{\pi }\left(t\right):=\int_{t}^{\infty }r\left(s^{\pi }\left(t^{\prime }\right)\right)e^{-\frac{t^{\prime }-t}{\tau_{r}}}dt^{\prime }. \end{array}$$

Here, *s*(*t*) denotes the state of the agent at time *t*, *r*(*s*^π^(*t*)) denotes the reward obtained in state *s*(*t*), τ_*r*_ a discounting factor for future rewards and π the agent's policy. To achieve this, we define the following objective function which should be minimized by gradient descent on the weights from inputs to the critic:

(4) $$\begin{array}{l} E\left(t\right):=\frac{1}{2}{\left(V^{\pi }\left(t\right)-z\left(t\right)\right)}^{2}, \end{array}$$

where *z*(*t*) represents the activity of the critic unit. By performing gradient descent on Equation (4), using a self-consistency equation for *V*^π^(*t*) from the derivative of Equation (3) and bootstrapping on the current prediction for the value (see Supplementary Material and Doya, 2000; Frémaux et al., 2013), we obtain the following local Hebbian three-factor learning rule that approximately minimizes the objective function (Equation 4):

(5) $$\begin{array}{l} \Delta w_{j}=\eta \delta \left(t\right)x_{j}\left(t\right)\Theta \left(z\left(t\right)-\theta_{\text{post}}\right), \end{array}$$

where η is a learning rate, *x*_*j*_(*t*) represents the activity of the *j*th place cell, Θ(·) the Heaviside function and θ_post_ a parameter that accounts for noise on the postsynaptic unit (see Supplementary Material for details). The term $\delta \left(t\right)=\dot{v}\left(t\right)+r\left(t\right)-\frac{1}{\tau_{r}}v\left(t\right)$ corresponds to the activity of the reward prediction error unit, acting as a neuromodulatory signal for the Hebbian plasticity between the presynaptic (*x*_*j*_) and postsynaptic (*z*) units. To avoid explicit calculation of the derivative, we approximate δ(*t*) by:

(6) $$\begin{array}{l} \delta \left(t\right)\approx \left(\frac{1}{d}-\frac{1}{\tau_{r}}\right)v\left(t\right)-\frac{1}{d}v\left(t-d\right)+r\left(t\right). \end{array}$$

To compute the derivative we hence implement two connections from the critic to the reward-prediction error unit: one instantaneous, and one with delay *d* > 0.

As proposed by Frémaux et al. (2013), to learn an optimal policy, we exploit that the actor units follow the same dynamics as the critic. We hence apply the same learning rule to the connections between the inputs and the actor units. In order to assure that at least one actor unit is active, thus preventing a deadlock, we introduce a minimal weight for each connection between input and output units and add input noise to the actor units.

#### 3.3.2. Mountain Car

**Figure 3:**
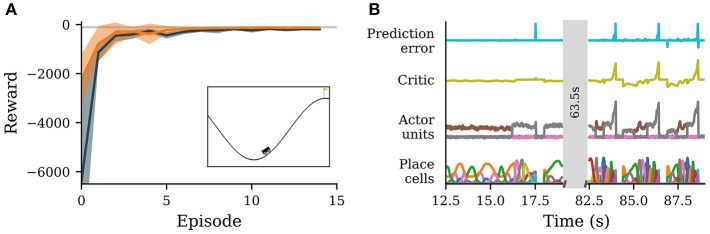
A neuronal network simulated in NEST successfully learns to navigate in an environment with continuous states and discrete actions. **(A)** Reward obtained by the agent per episode averaged over 10 simulations with different seeds (solid orange curve). Orange band indicates ± one standard deviation. Dark gray represents the reward obtained from Q-learning. The light gray line marks average reward per episode for which the environment is considered solved. Inset: screenshot of the environment with agent (stylized vehicle), environment with valley and two hills and goal position (yellow flag). The agent is close to a typical starting position at the trough. **(B)** Activity traces of place cells (bottom), actor units (second from bottom), critic unit (second from top) and reward prediction error unit (top). Shown are neural activities during 6.5 s early (left) and late (right) during learning. The neural network simulation was run with a real-time factor of one.

As an example of an environment with continuous states, we consider the *MountainCar*^8^ environment. The task is to steer a toy vehicle that starts at a valley between two hills to the top of the right one (Figure 3A, inset). To make the task more challenging, the car's engine is not strong enough to reach the top in one go, so the agent needs to learn to gain momentum by swinging back and forth between the two hills. A single episode in this environment starts when the agent is placed in the valley and ends when it reaches the final position on the top of the right hill. The state of the agent is described by two continuous variables: the x-position *x*(*t*) and the x-velocity ẋ(*t*). The agent can choose from three different discrete actions that affect the velocity of the vehicle (accelerate left, no acceleration, accelerate right). It receives punishment (i.e., negative reward) from the environment in every step; the goal is to minimize the total punishment collected over the whole episode. Since it is challenging for a neuronal network implementation of the actor-critic architecture with exclusively excitatory synapses to learn the value function corresponding to a task with solely negative reinforcement (Potjans et al., 2011), we provide additional reward when the agent reaches the final position.

To translate the agent's current state into neuronal activity, we distribute 25 place cells evenly across the two-dimensional plane of possible positions and velocities using the *discretize adapter* of the RMT. The actor is implemented by a WTA circuit of three units as shown in (3.3.1). The activity of these units is transformed into an action via the *argmax adapter* (3.1).

We compare the performance of our neuronal network to Q-learning (Watkins and Dayan, 1992) with function approximation via a multi-layer perceptron (see e.g., Tesauro, 1995; Mnih et al., 2013). The position and velocity of the car are projected to a population of hidden units with rectifying-linear activation function, which in turn project to three output units, encoding the estimated Q-value of each possible action. These Q-values are used by an epsilon-greedy strategy to select the next move. We use the ADAM optimizer (Kingma and Ba, 2014) and memory replay (Lin, 1993) to train the Q-function network (see Supplementary Material for details).

Initially, the agent explores the environment by selecting random actions. Due to the WTA circuit dynamics, a single actor neuron stays active over an extended period of time. The constant punishment gradually decreases the weights from the place cells to the corresponding actor unit, eventually leading to another actor unit becoming active (Figure 3B, left). After a while, the agent reaches the goal by performing actions that have not been significantly punished. For this task the stable nature of the WTA is advantageous, causing the agent to perform the same action repeatedly allowing efficient exploration of the state space. After the agent has found the goal once, the number of steps spent on exploring actions in the following episodes is much smaller. From the sixth episode on, the performance of the agent is already close to optimal (Figure 3A). After learning for about ten episodes, the agent's performance has converged. The value of the final state has been successfully propagated backwards over different states, leading to a ramping of activity of the critic unit from the start of an episode to the end (Figure 3B, right).

In comparison to Q-learning, the agent avoids high losses at the start of a training episode. This can most likely be traced back to two factors, which endow our agent with an advantage over Q-learning with function approximation. First, our agent starts with predefined place cells that reliably encode the position in state space and it only has to learn to appropriately combine the activities of these place cells. In contrast, Q-learning starts from a completely blank slate, with no prior knowledge about the input space. It would be incorrect to conclude from this that place cells are generally the superior strategy: manually-defined place cells become infeasible in high-dimensional state spaces as their number increases exponentially in the number of input-space dimensions, whereas Q-learning with function approximation can be scaled to very high-dimensional input spaces (see e.g., Mnih et al., 2013). The second advantage of our agent are long transients in action selection. Before learning the correct sequence of actions, the agent tends to explore a single action for an extended period of time (see trajectories of actor units, Figure 3B, left), whereas Q-learning changes action often. For this particular environment sticking to one action for an extended period of time, especially during the early phases of learning, is advantageous as the final strategy involves few action changes (Figure 3B, right). This disadvantage can most likely be attenuated by using frame skipping or similar methods (cf. Mnih et al., 2013).

#### 3.3.3. Frozen Lake

**Figure 4:**
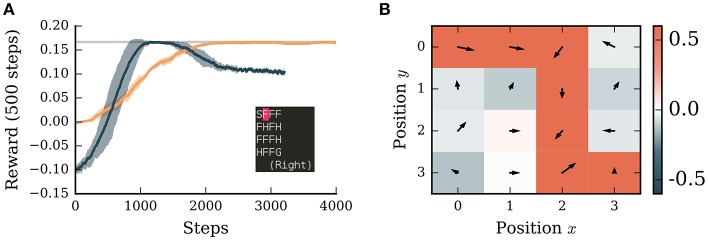
A neuronal network simulated in NEST successfully learns to navigate in a grid world with discrete states and discrete actions. **(A)** Orange curve: average reward collected by the agent over the next 500 steps averaged over 5 simulations. Dark blue curve: performance of Potjans et al. (2011) model averaged over 5 simulations. Shaded bands indicates ± one standard deviation. Gray line: theoretical optimum. Inset: screenshot of the environment with start state (S), frozen states (F), holes (H), and goal state (G). The position of the agent is indicated in pink. **(B)** The learned policy and value map of the environment. Red colors indicate positive, blue colors negative values. Arrows indicate the preferred direction of movement. The neural network simulation was run with a real-time factor of two.

As a second application illustrating the use of the toolchain for discrete environments, we train the same network model on the *FrozenLake*^9^ environment. This consists of a discrete set of 16 states arranged in a four-by-four grid (Figure 4A, inset). Each state is either a start state (S), a goal state (G), a hole (H), or a frozen state (F). From the start position, the agent has to reach the rewarded state by navigating over the frozen states without falling into holes which reset the agent to the starting position. In each step the agent can choose from four different actions: move west, move north, move east and move south. Usually, the tiles are “slippery,” i.e., there is a chance that a random action is executed irrespective of the action chosen by the agent. However, to simplify learning for demonstration purposes we turn this feature off. Upon reaching the goal the agent receives a reward of magnitude one. Since the optimal path involves six steps from start to goal, the theoretical optimal reward per step is ~0.16. To encourage exploration the agent receives a small punishment in each state and, additionally, to speed up learning the agent is punished for falling into holes.

Unlike in the continuous *MountainCar* environment, the tuning curves of place cells do not overlap in the discrete case, leading to sharp transitions in the network activity. This leads to severe issues for associating values and actions with the respective states. To address this problem we introduced a simple eligibility trace by evaluating the activity of the pre- and post synaptic units in the learning rule with a small delay δ*t* (see Supplementary Material). With this addition, the network model is able to find the optimal solution for this task within roughly 2,000 steps (Figure 4A). It also learns to associate holes with punishment and frozen states with reward if they are on the path to the goal (Figure 4B). Although there are two possible paths to the goal, the agent prefers the path with fewer corners, likely as a consequence of the WTA circuit which tends to select the same action repeatedly.

We compare the performance of our algorithm to an adapted spiking neural network model of the basal ganglia implementing reinforcement learning (Potjans et al., 2011; Jitsev et al., 2012). Learning in this algorithm is faster than our implementation and reaches the optimal solution after only 1,000 steps (Figure 4A). However, the performance of the spiking model drops after 2,000 steps to a sub-optimal value. As this model relies on a very high discount factor (γ = 0.99), which is close to 'infinite horizon', the values of the states saturate in the vicinity to the goal. This can lead to a low contrast of preferred actions in those states and therefore to a sub-optimal policy. To resolve this issue is beyond the scope of this manuscript (see Kato and Morita, 2016 for an investigation of such matters), but underlines the importance of comparing alternative models on the same task. Only through such activities can we identify the strengths and weaknesses of different functional hypotheses and thus make more rapid progress in the field.

## 4. Conclusion

In this manuscript, we have argued that standardized benchmarks are of critical importance to compare and improve functional neural network models. Moreover, to investigate the characteristics of the neural circuits that allow agents to operate autonomously in their environments and learn appropriate behaviors, simulation infrastructure must enable closed-loop interaction between agent and environment.

To make such a set of closed-loop benchmarks available to the computational neuroscience community, we have developed a toolchain that closes the loop between the OpenAI Gym and neural network simulators implementing the MUSIC interface, notably NEST and NEURON. We demonstrated the functionality of the toolchain by implementing an actor-critic architecture in NEST and evaluating its performance on two different environments. The performance of the network quickly reached near-optimal performance on these two tasks.

Compared to creating customized environments within the framework of a neuronal simulator, using readily available, well-tested tools is considerably easier (and thus faster) for the researcher, often computationally more efficient, and most importantly, supports reproducible science. In addition, having the OpenAI Gym environments as common benchmarks in both fields encourages comparison between traditional machine learning and biologically plausible implementations. In contrast to models presented in previous studies, our toolchain makes it easy for other researchers to extend our implementation of an actor-critic architecture to other environments, replace neuron models or explore alternative learning rules. The simulation and visualization scripts to reproduce the results presented for the network model described here are publicly available^10^, and so can serve as a starting point for more complex models. In addition a dedicated tutorial introduces the toolchain step-by-step using NEST as an example simulator^11^.

While the toolchain currently only supports the OpenAI Gym, the extension to other toolkits is simple due to a modular design of the wrapper. The RMT can be found on GitHub and is available under the GPLv3. The OpenAI Gym ZeroMQ wrapper is also available via GitHub under the MIT license. A complementary development to the work presented here is provided by SPORE, a framework for reward-based learning with spiking neurons in the NEST simulator^12^. It provides support for synapse models with time-driven updates, additional support for recording and evaluating traces of neuronal state variables and introduces MUSIC ports for communicating rewards to a running simulation.

With the work presented here we enable researchers to build more easily upon previous studies and evaluate novel models. We hope this boosts the progress in computational neuroscience in uncovering the biophysical mechanisms involved in autonomous behavior and learning.

## Data Availability

The datasets generated for this study can be found on GitHub (https://github.com/INM-6/closed-loop-learning-in-autonomous-agents).

## Author Contributions

This study was conceived and designed by JJ, PW, and AM. The toolchain and tested models were implemented by JJ and PW. The simulations were carried out and analyzed by JJ and PW. The manuscript was written jointly by all authors.

### Conflict of Interest Statement

The authors declare that the research was conducted in the absence of any commercial or financial relationships that could be construed as a potential conflict of interest.

## Footnotes

^1^https://gym.openai.com/envs

^2^http://nest-simulator.org/

^3^https://nest-initiative.org/

^4^https://gym.openai.com/envs/#atari

^5^https://gym.openai.com/envs/#mujoco

^6^https://github.com/incf-music/ros-music-adapters

^7^https://github.com/incf-music/music-adapters

^8^https://gym.openai.com/envs/MountainCar-v0/

^9^https://gym.openai.com/envs/FrozenLake-v0/

^10^https://github.com/INM-6/closed-loop-learning-in-autonomous-agents

^11^https://github.com/INM-6/nestrl-tutorial/

^12^https://github.com/IGITUGraz/spore-nest-module

## References

[B1] Abadi M. Agarwal A. Barham P. Brevdo E. Chen Z. Citro C. (2016). Tensorflow: Large-scale machine learning on heterogeneous distributed systems. arXiv preprint arXiv:1603.04467.

[B2] Aswolinskiy W. Pipa G. (2015). RM-SORN: a reward-modulated self-organizing recurrent neural network. Front. Comput. Neurosci. 9:36. 10.3389/fncom.2015.00036 25852533 PMC4371712

[B3] Baladron J. Hamker F. H. (2015). A spiking neural network based on the basal ganglia functional anatomy. Neural Netw. 67, 1–13. 10.1016/j.neunet.2015.03.002 25863288

[B4] Bekolay T. Bergstra J. Hunsberger E. DeWolf T. Stewart T. C. Rasmussen D.. (2013). Nengo: a python tool for building large-scale functional brain models. Front. Neuroinform. 7:48. 10.3389/fninf.2013.00048 24431999 PMC3880998

[B5] Bellemare M. Naddaf Y. Veness J. Bowling M. (2015). The arcade learning environment: an evaluation platform for general agents, in Twenty-Fourth International Joint Conference on Artificial Intelligence.

[B6] Bengio Y. Lamblin P. Popovici D. Larochelle H. (2007). Greedy layer-wise training of deep networks. Adv. Neural Inform. Process. Syst. 19:153.

[B7] Bower J. M. Beeman D. (2007). GENESIS (simulation environment). Scholarpedia 2:1383 10.4249/scholarpedia.1383

[B8] Brockman G. Cheung V. Pettersson L. Schneider J. Schulman J. Tang J. (2016). OpenAI Gym. ArXiv e-prints. arXiv preprint arXiv:1606.01540.

[B9] Carnevale T. Hines M. (2006). The NEURON Book. Cambridge: Cambridge University Press.

[B10] Collobert R. Bengio S. Mariéthoz J. (2002). Torch: A Modular Machine Learning Software Library. Technical report, Idiap.

[B11] Deng J. Dong W. Socher R. Li L.-J. Li K. Fei-Fei L. (2009). ImageNet: a large-scale hierarchical image database, in CVPR09.

[B12] Djurfeldt M. Hjorth J. Eppler J. M. Dudani N. Helias M. Potjans T. C.. (2010). Run-time interoperability between neuronal simulators based on the music framework. Neuroinformatics 8, 43–60. 10.1007/s12021-010-9064-z 20195795 PMC2846392

[B13] Doya K. (2000). Reinforcement learning in continuous time and space. Neural Comput. 12, 219–245. 10.1162/089976600300015961 10636940

[B14] Echeverria G. Lassabe N. Degroote A. Lemaignan S. (2011). Modular open robots simulation engine: Morse, in 2011 IEEE International Conference on Robotics and Automation (Citeseer), 46–51.

[B15] Eppler J. M. Helias M. Muller E. Diesmann M. Gewaltig M. (2009). PyNEST: a convenient interface to the NEST simulator. Front. Neuroinform. 2:12. 10.3389/neuro.11.012.2008 19198667 PMC2636900

[B16] Falotico E. Vannucci L. Ambrosano A. Albanese U. Ulbrich S. Vasquez Tieck J. C.. (2017). Connecting artificial brains to robots in a comprehensive simulation framework: the neurorobotics platform. Front. Neurorobot. 11:2. 10.3389/fnbot.2017.00002 28179882 PMC5263131

[B17] Frémaux N. Sprekeler H. Gerstner W. (2010). Functional requirements for reward-modulated spike-timing-dependent plasticity. J. Neurosci. 30, 13326–13337. 10.1523/JNEUROSCI.6249-09.2010 20926659 PMC6634722

[B18] Frémaux N. Sprekeler H. Gerstner W. (2013). Reinforcement learning using a continuous time actor-critic framework with spiking neurons. PLoS Comput. Biol. 9:e1003024. 10.1371/journal.pcbi.1003024 23592970 PMC3623741

[B19] Friedrich J. Lengyel M. (2016). Goal-directed decision making with spiking neurons. J. Neurosci. 36, 1529–1546. 10.1523/JNEUROSCI.2854-15.2016 26843636 PMC4737768

[B20] Friedrich J. Urbanczik R. Senn W. (2014). Code-specific learning rules improve action selection by populations of spiking neurons. Int. J. Neural Syst. 24:1450002. 10.1142/S0129065714500026 24875790

[B21] Gerstner W. Naud R. (2009). How good are neuron models? Science 326, 379–380. 10.1126/science.1181936 19833951

[B22] Gewaltig M.-O. Diesmann M. (2007). NEST (NEural Simulation Tool). Scholarpedia 2:1430 10.4249/scholarpedia.1430

[B23] Goodman D. F. Brette R. (2009). The brian simulator. Front. Neurosci. 3, 192–197. 10.3389/neuro.01.026.2009 20011141 PMC2751620

[B24] Hahne J. Dahmen D. Schuecker J. Frommer A. Bolten M. Helias M.. (2017). Integration of continuous-time dynamics in a spiking neural network simulator. Front. Neuroinform. 11:34. 10.3389/fninf.2017.00034 28596730 PMC5442232

[B25] Hazan H. Saunders D. J. Khan H. Patel D. Sanghavi D. T. Siegelmann H. T. Kozma R. (2018). Bindsnet: a machine learning-oriented spiking neural networks library in python. Front. Neuroinform. 12:89. 10.3389/fninf.2018.00089 30631269 PMC6315182

[B26] Hintjens P. (2013). ZeroMQ: Messaging for Many Applications. O'Reilly Media, Inc.

[B27] Hinton G. Deng L. Yu D. Dahl G. E. Mohamed A.-R. Jaitly N. (2012). Deep neural networks for acoustic modeling in speech recognition: the shared views of four research groups. IEEE Signal Process. Mag. 29, 82–97. 10.1109/MSP.2012.2205597

[B28] Hinton G. E. Salakhutdinov R. R. (2006). Reducing the dimensionality of data with neural networks. Science 313, 504–507. 10.1126/science.1127647 16873662

[B29] Hinton G. E. Srivastava N. Krizhevsky A. Sutskever I. Salakhutdinov R. R. (2012). Improving neural networks by preventing co-adaptation of feature detectors. arXiv preprint arXiv:1207.0580.

[B30] Human Brain Project (2014). Project Website. Available online at: http://www.humanbrainproject.eu

[B31] Izhikevich E. M. (2007). Solving the distal reward problem through linkage of STDP and dopamine signaling. Cereb. Cortex 17, 2443–2452. 10.1093/cercor/bhl152 17220510

[B32] James B. Olivier B. Frédéric B. Pascal L. Razvan P. (2010). Theano: a CPU and GPU math expression compiler, in Proceedings of the Python for Scientific Computing Conference (SciPy).

[B33] Jia Y. Shelhamer E. Donahue J. Karayev S. Long J. Girshick R. (2014). Caffe: convolutional architecture for fast feature embedding. arXiv preprint arXiv:1408.5093. 10.1145/2647868.2654889

[B34] Jitsev J. Morrison A. Tittgemeyer M. (2012). Learning from positive and negative rewards in a spiking neural network model of basal ganglia, in The 2012 International Joint Conference on Neural Networks (IJCNN) (IEEE), 1–8.

[B35] Jordan J. Ippen T. Helias M. Kitayama I. Sato M. Igarashi J. (2018). Extremely scalable spiking neural network simulation code: from laptops to exascale computers. Front. Neuroinform. 12:2 10.3389/fninf.2018.00034 29503613 PMC5820465

[B36] Kato A. Morita K. (2016). Forgetting in reinforcement learning links sustained dopamine signals to motivation. PLoS Comput. Biol. 12:e1005145. 10.1371/journal.pcbi.1005145 27736881 PMC5063413

[B37] Kingma D. P. Ba J. (2014). Adam: a method for stochastic optimization. arXiv preprint arXiv:1412.6980.

[B38] Koenig N. Howard A. (2004). Design and use paradigms for gazebo, an open-source multi-robot simulator, in 2004 IEEE/RSJ International Conference on Intelligent Robots and Systems (IROS)(IEEE Cat. No. 04CH37566), Vol. 3 (IEEE), 2149–2154.

[B39] Krizhevsky A. Hinton G. (2009). Learning Multiple Layers of Features From Tiny Images. Technical Report 4, University of Toronto.

[B40] Krizhevsky A. Sutskever I. Hinton G. E. (2012). Imagenet classification with deep convolutional neural networks, in Advances in Neural Information Processing Systems, 1097–1105.

[B41] Kunkel S. Schmidt M. Eppler J. M. Plesser H. E. Masumoto G. Igarashi J. Ishii S.. (2014). Spiking network simulation code for petascale computers. Front. Neuroinform. 8:78. 10.3389/fninf.2014.00078 25346682 PMC4193238

[B42] LeCun Y. (1998). The MNIST Database of Handwritten Digits. Available online at: http://yann.lecun.com/exdb/mnist

[B43] Lin L.-J. (1993). Reinforcement Learning for Robots Using Neural Networks. Technical report, Carnegie-Mellon Univ Pittsburgh PA School of Computer Science.

[B44] Michel O. (2004). Cyberbotics Ltd. Webots™ : professional mobile robot simulation. Int. J. Adv. Robot. Syst. 1:5 10.5772/5618

[B45] Mnih V. Badia A. P. Mirza M. Graves A. Lillicrap T. Harley T. (2016). Asynchronous methods for deep reinforcement learning, in Int. Conf. Mach. Learn. 1928–1937.

[B46] Mnih V. Kavukcuoglu K. Silver D. Graves A. Antonoglou I. Wierstra D. (2013). Playing atari with deep reinforcement learning. arXiv preprint arXiv:1312.5602.

[B47] Mnih V. Kavukcuoglu K. Silver D. Rusu A. A. Veness J. Bellemare M. G.. (2015). Human-level control through deep reinforcement learning. Nature 518, 529–533. 10.1038/nature14236 25719670

[B48] Moren J. Sugimoto N. Doya K. (2015). Real-time utilization of system-scale neuroscience models. J. Jap. Neural Netw. Soc. 22, 125–132. 10.3902/jnns.22.125

[B49] Moser E. I. Kropff E. Moser M.-B. (2008). Place cells, grid cells, and the brain's spatial representation system. Annu. Rev. Neurosci. 31, 69–89. 10.1146/annurev.neuro.31.061307.090723 18284371

[B50] Paszke A. Gross S. Chintala S. Chanan G. Yang E. DeVito Z. (2017). Automatic Differentiation in Pytorch.

[B51] Potjans W. Diesmann M. Morrison A. (2011). An imperfect dopaminergic error signal can drive temporal-difference learning. PLoS Comput. Biol. 7:e1001133. 10.1371/journal.pcbi.1001133 21589888 PMC3093351

[B52] Potjans W. Morrison A. Diesmann M. (2009). A spiking neural network model of an actor-critic learning agent. Neural Comput. 21, 301–339. 10.1162/neco.2008.08-07-593 19196231

[B53] Quigley M. Conley K. Gerkey B. Faust J. Foote T. Leibs J. (2009). ROS: an open-source Robot Operating System, in ICRA Workshop on Open Source Software, Vol. 3 (Kobe).

[B54] Raina R. Madhavan A. Ng A. Y. (2009). Large-scale deep unsupervised learning using graphics processors, in Proceedings of the 26th Annual International Conference on Machine Learning (ACM), 873–880.

[B55] Rasmussen D. Eliasmith C. (2014). A neural model of hierarchical reinforcement learning, in CogSci. 10.1371/journal.pone.0180234 PMC5500327 28683111

[B56] Rombouts J. o. Bohte S. M. Roelfsema P. R. (2015). How attention can create synaptic tags for the learning of working memories in sequential tasks. PLoS Comput. Biol. 11:e1004060. 10.1371/journal.pcbi.1004060 25742003 PMC4351255

[B57] Rueckert E. Kappel D. Tanneberg D. Pecevski D. Peters J. (2016). Recurrent spiking networks solve planning tasks. Sci. Rep. 6:21142. 10.1038/srep21142 26888174 PMC4758071

[B58] Russakovsky O. Deng J. Su H. Krause J. Satheesh S. Ma S. (2015). ImageNet large scale visual recognition challenge. Int. J. Comput. Vis. 115, 211–252. 10.1007/s11263-015-0816-y

[B59] Sanz Leon P. Knock S. A. Woodman M. M. Domide L. Mersmann J. McIntosh A. R.. (2013). The virtual brain: a simulator of primate brain network dynamics. Front. Neuroinform. 7:10. 10.3389/fninf.2013.00010 23781198 PMC3678125

[B60] Sheik S Liu Q. (2019). SINABS - A Spiking Deep Neural Network Inference Emulator. 10.5281/zenodo.3218477

[B61] Silver D. Huang A. Maddison C. J. Guez A. Sifre L. Van Den Driessche G.. (2016). Mastering the game of go with deep neural networks and tree search. Nature 529, 484–489. 10.1038/nature16961 26819042

[B62] Sutton R. S. Barto A. G. (1998). Reinforcement Learning: An Introduction. Adaptive Computation and Machine Learning. The MIT Press.

[B63] Tesauro G. (1995). Temporal difference learning and TD-gammon. Commun. ACM 38, 58–68.

[B64] Todorov E. Erez T. Tassa Y. (2012). Mujoco: a physics engine for model-based control, in 2012 IEEE/RSJ International Conference on Intelligent Robots and Systems (IROS), 5026–5033.

[B65] Urbanczik R. Senn W. (2009). Reinforcement learning in populations of spiking neurons. Nat. Neurosci. 12:250. 10.1038/nn.2264 19219040

[B66] Vasilaki E. Frémaux N. Urbanczik R. Senn W. Gerstner W. (2009). Spike-based reinforcement learning in continuous state and action space: when policy gradient methods fail. PLoS Comput. Biol. 5:e1000586. 10.1371/journal.pcbi.1000586 19997492 PMC2778872

[B67] Watkins C. J. Dayan P. (1992). Q-learning. Mach. Learn. 8, 279–292.

[B68] Watkins C. J. C. H. (1989). Learning from delayed rewards (Ph.D. thesis). University of Cambridge.

[B69] Weidel P. Djurfeldt M. Duarte R. C. Morrison A. (2016). Closed loop interactions between spiking neural network and robotic simulators based on music and ros. Front. Neuroinform. 10:31. 10.3389/fninf.2016.00031 27536234 PMC4971076

[B70] Wils S. De Schutter E. (2009). STEPS: modeling and simulating complex reaction-diffusion systems with Python. Front. Neuroinform. 3:15. 10.3389/neuro.11.015.2009 19623245 PMC2706651

[B71] Wilson M. (2002). Six views of embodied cognition. Psychon. Bull. Rev. 9, 625–636. 10.3758/BF03196322 12613670

