# Supplementary material for: A Closed-Loop Toolchain for Neural Network Simulations of Learning Autonomous Agents
Source: Front Comput Neurosci. 2019 Aug 2;13:46. doi: 10.3389/fncom.2019.00046 (PMC6687756; doi:10.3389/fncom.2019.00046)
Supplement: Supplementary file 1 [file Data_Sheet_1.PDF]

frontiers

Supplementary Material

1 DERIVATION OF THE LEARNING RULE

We consider continuous time, continuous states and continuous actions and follow similar steps as Doya (2000); Frémaux et al. (2013). Starting from the continuous-time value function

$$V^{\pi}(\mathbf{s}(t)) := \int_{t}^{\infty} r(\mathbf{s}^{\pi}(t'))e^{-\frac{t'-t}{\tau_r}}\, dt' \,, \tag{S1}$$

we take the derivative with respect to t to arrive at a self-consistency equation for V:

$$\dot{V}^{\pi}(t) := \frac{dV^{\pi}(t)}{dt} = -r(t) + \frac{1}{\tau_r}V^{\pi}(t)\,. \tag{S2}$$

To implement temporal-difference learning in a neural network architecture, we would like to approximately represent the true value $V^{\pi}(t)$ of the state at time t by the rate $z_i(t)$ of a critic neuron. This activity will depend on the activity of input units, i.e., place cells, and the weights between inputs and critic. With initially random weights the self-consistency criterion will not be fulfilled, and will have a finite error $\delta(t)$:

$$\delta(t) = \dot{z}_i(t) + r(t) - \frac{1}{\tau_r}z_i(t)\,. \tag{S3}$$

We now define an objective function that should be minimized by gradient descent on the weights:

$$E(t) = \frac{1}{2}(V^{\pi}(t) - z_i(t))^2\,. \tag{S4}$$

We take derivative with respect to w_{ij} and use the self-consistency equation (S2):

$$\begin{aligned}\frac{\partial E(t)}{\partial w_{ij}} &= -(V^{\pi}(t) - z_i(t))\frac{\partial z_i(t)}{\partial w_{ij}} \\ &= -(\tau_r\dot{V}^{\pi}(t) + \tau_r r(t) - z_i(t))\frac{\partial z_i(t)}{\partial w_{ij}} \\ &\approx -\underbrace{(\tau_r\dot{z}(t) + \tau_r r(t) - z_i(t))}_{\tau_r\delta(t)}\frac{\partial z_i(t)}{\partial w_{ij}}\end{aligned} \tag{S5}$$

Here we have replaced $\dot{V}^{\pi}(t)$ with $\dot{z}_i(t)$ in the last line. For a discussion of the validity of this approximation and the convergence of the learning rule, see Frémaux et al. (2013). To perform gradient descent on the objective function, we hence need to change the weights according to

$$\begin{aligned}\Delta w_{ij} &:= -\,\eta'\frac{\partial E(t)}{\partial w_{ij}} \\ &=\eta\delta(t)\frac{\partial z_i(t)}{\partial w_{ij}}\,,\end{aligned} \tag{S6}$$

where we introduced $\eta = \eta' \tau_r$. We hence need to determine the derivative of the critic activity with respect to the weights between inputs and critic $\frac{\partial z_i(t)}{\partial w_{ij}}$.

We start from the differential equation describing the dynamics of a threshold-linear rate neuron, and assume the noise to be small, i.e., we drop the term $\xi_i(t)$. Without loss of generality, we assume $\mu_i = 0, \theta_i = 0$. The dynamics are then given by the solution to

$$\tau \frac{dz_i(t)}{dt} = -z_i(t) + g\,\Theta\left(\sum_j w_{ij} x_j(t)\right)\left(\sum_j w_{ij} x_j(t)\right) . \tag{S7}$$

Variation of constants yields the general solution as a convolution equation:

$$z_i(t) = g\left(\left(\Theta\left(\ldots\right)\left(\sum_j w_{ij} x_j(\cdot)\right)\right) * \kappa(\cdot)\right)(t)\,, \tag{S8}$$

where we have introduced the filter kernel $\kappa(t) = \frac{1}{\tau} e^{-\frac{t}{\tau}} \Theta(t)$. We now take the derivative with respect to w_{ij}. While the derivative at $\sum_j w_{ij} x_j(t) = 0$ is technically not defined, we follow the usual convention and set it to zero. This yields

$$\frac{\partial z_i(t)}{\partial w_{ij}} = \begin{cases} g\left(x_j * \kappa\right)(t) & \text{if } \sum_j w_{ij} x_j(t) > 0 \\ 0 & \text{else} \end{cases} \tag{S9}$$

By combining equation (S6) with equation (S9), we obtain the following learning rule:

$$\Delta w_{ij} = \begin{cases} \eta \delta(t) g (x_j * \kappa)(t) & \text{if } \sum_j w_{ij} x_j(t) > 0 \\ 0 & \text{else} \end{cases} \tag{S10}$$

By choosing the time constant of critic and actors small, we effectively remove the filtering of the presynaptic activity ($\lim_{\tau \to 0} \kappa(t) = \delta(t)$) and hence ignore it. To simplify this equation further, we rewrite it as a condition on the rate of the postsynaptic neuron by observing that $z_i(t) > 0$ iff $\sum_j w_{ij} x_j(t) > 0$. To implement exploration for similar inputs to all output units we add noise to the activity of the actor units. We only consider a postsynaptic neuron active, if its activity is larger than some threshold θ_{post}. This leads to the following form for the learning rule:

$$\Delta w_{ij} = \eta \delta(t) g x_j(t) \Theta(z_i(t) - \theta_{\text{post}})\,, \tag{S11}$$

where we $\Theta(\cdot)$ denotes the Heaviside step function defined as:

$$\Theta(x) = \begin{cases} 1 & x > 0 \\ 0 & \text{else} \end{cases}$$

To implement a simple type of eligibility trace, we introduce an additional parameter δt that can delay the activity of the pre- and post-synaptic units in the learning rule:

$$\Delta w_{ij} = \eta \delta(t) g x_j(t - \delta t) \Theta(z_i(t - \delta t) - \theta_{\text{post}})\,, \tag{S12}$$

2 JSON MESSAGE TYPES

Listing 1 show the standard message types used for communication between the OpenAI Gym and the RMT. All messages are serialized using JSON and communicated via ZeroMQ.

Listing 1. Message types used for communication.

```
BasicMsg
{
    float min
    float max
    float value
    float timestamp
}

ObservationMsg
{
    BasicMsg[] observations   # one basic msg per dimension
}

RewardMsg
{
    BasicMsg[] reward         # reward is always one dimensional
}

ActionMsg
{
    BasicMsg[] actions        # one dimensional for discrete actions
                              # or one dimension per possible action
}
```

3 EXAMPLE WRAPPER CONFIGURATION FILE

Listing 2 shows an example configuration file for running the mountain car environment.

Listing 2. Example configuration file for the wrapper to run the "MountainCar-v0" environment.

```
"All":
{
    "seed": 12345,
    "time_stamp_tolerance": 0.01,
    "prefix": null,
    "write_report": true,
    "report_file": "./report.json",
    "overwrite_files": false,
    "flush_report_interval": null
},
"Env":
{
    "env": "MountainCar-v0",
    "initial_reward": null,
    "final_reward": null,
    "min_reward": -1.0,
    "max_reward": 1.0,
    "render": true,
    "monitor": false,
    "monitor_dir": "./experiment-0/",
    "monitor_args":
    {
        "write_upon_reset": true,
        "video_callable": false
    }
},
"EnvRunner":
{
    "update_interval": 0.01,
    "inter_trial_duration": 0.4
},
"CommandReceiver":
{
    "socket": 5555,
    "time_stamp_tolerance": 0.01
},
"ObservationSender":
{
    "socket": 5556,
    "update_interval": 0.01
},
"RewardSender":
{
    "socket": 5557,
    "update_interval": 0.01
}
```

4 EXAMPLE MUSIC CONFIGURATION FILE

Listing 3 shows an example MUSIC configuration file to run the MountainCar environment. It shows the different processes with parameters which are spawned by MUSIC including RMT adapters and NEST.

Listing 3. Example MUSIC configuration file to run the MountainCar environment.

```
stoptime=150.
rtf=1.
[reward]
  binary=zmq_in_adapter
  args=
  np=1
  music_timestep=0.001
  message_type=GymObservation
  zmq_topic=
  zmq_addr=tcp://localhost:5557
[sensor]
  binary=zmq_in_adapter
  args=
  np=1
  music_timestep=0.001
  message_type=GymObservation
  zmq_topic=
  zmq_addr=tcp://localhost:5556
[discretize]
  binary=discretize_adapter
  args=
  np=1
  music_timestep=0.001
  grid_positions_filename=grid_pos.json
[nest]
  binary=../actor_critic_network/network.py
  args=-t 150. -n 25 -m 3 -p network_params.json
  np=1
[argmax]
  binary=argmax_adapter
  args=
  np=1
  music_timestep=0.001
[command]
  binary=zmq_out_adapter
  args=
  np=1
  music_timestep=0.01
  message_type=GymCommand
  zmq_topic=
  zmq_addr=tcp://*:5555
sensor.out->discretize.in[2]
discretize.out->nest.in[25]
reward.out->nest.reward_in[1]
nest.out->argmax.in[3]
argmax.out->command.in[1]
```

5 NETWORK DESCRIPTION

The tables S1, S2, S3 summarize the network architecture and parameters.

6 ENVIRONMENTS

Table S4 shows parameters for the OpenAI Gym environments and the ZeroMQ wrapper.

REFERENCES

Doya, K. (2000). Reinforcement learning in continuous time and space 12, 219–245

Frémaux, N., Sprekeler, H., and Gerstner, W. (2013). Reinforcement learning using a continuous time actor-critic framework with spiking neurons. *PLoS Comput Biol* 9, e1003024

Nordlie, E., Gewaltig, M.-O., and Plesser, H. E. (2009). Towards reproducible descriptions of neuronal network models 5, e1000456

A	Model summary
Populations	Seven
Topology	None
Connectivity	Population specific
Neuron model	Linear & threshold-linear rate
Channel models	None
Synapse model	Instantaneous & delayed continuous coupling
Plasticity	Three-factor Hebbian
External input	Continuous MUSIC ports
External output	Continuous MUSIC ports
Measurements	Rates of all neurons

B	Populations	
**Name**	**Elements**	**Size**
Observation	MUSIC in port	1
Reward	MUSIC in port	1
Action	MUSIC out port	1
Place cells	Threshold-linear	$16(25)$
Critic	Threshold-linear	1
Actor	Threshold-linear	$4(3)$
Prediction error	Linear	1

C	Connectivity	
**Source**	**Target**	**Pattern**
Observation	Place cells	One-to-one (by MUSIC channel), instantaneous, static, weight w_{o}
Reward	Prediction error	One-to-one (by MUSIC channel), instantaneous, static, weight w_{r}
Actor	Action	One-to-one (by MUSIC channel), instantaneous, static, weight w_{a}
Place cells	Critic	All-to-all, instantaneous, plastic, initial weight w_{pc}
Place cells	Actor	All-to-all, instantaneous, plastic, initial weight w_{pa}
Critic	Prediction error	One-to-one, instantaneous, static, weight $1/d - 1/\tau_r$
Critic	Prediction error	One-to-one, delay d, static, weight $-1/d$
Actor	Actor	All-to-all, instantaneous, static, weight $\alpha \exp(-\Delta a/\sigma) + \beta$

D	Neuron and synapse model
Type	Linear rate neuron
Dynamics	$\tau \frac{dz(t)}{dt} = -z(t) + \mu + (h(t) - \theta) + \xi(t)$
Type	Threshold-linear rate neuron
Dynamics	$\tau \frac{dz(t)}{dt} = -z(t) + \mu + \Theta\left(h(t) - \theta\right)\left(h(t) - \theta\right) + \xi(t)$
Type	Three-factor Hebbian synapse
Plasticity	$\Delta w_{ij} = \eta \delta(t) g x_j(t - \delta t) \Theta(z_i(t - \delta t) - \theta_{\mathrm{post}})$

E	Input
**Type**	**Description**
Observation	Rate $r \in [-1, 1]$ according to tuning of place cell (using *discretize* adapter)
Reward	Rate $r \in [-1, 1]$ according to reward provided by the environment

F	Output
**Type**	**Description**
Action	Rates $r_i \in [0, \infty)$ according to activities of the actor units

Table S1. Description of the network model (according to Nordlie et al. (2009)).

B	Populations: place cells
**Name**	**Values**
τ	5.0 (1.0)
g	1.0
μ	0.0
σ_ξ	0.0
θ	-0.5

B	Populations: critic
**Name**	**Values**
τ	0.1
g	1.0
μ	-1.0
σ_ξ	0.0
θ	-1.0

B	Populations: reward
**Name**	**Values**
τ	1.0
g	1.0
μ	0.0
σ_ξ	0.0
θ	0.001 (-0.0999)

B	Populations: actor
**Name**	**Values**
τ	0.1
g	1.0
μ	0.0
σ_ξ	0.2 (0.05)
θ	0.0

Table S2. Table of the network parameters used for both tasks (according to Nordlie et al. (2009)). Values in brackets are used for the *MountainCar* environment.

C	Connectivity
**Name**	**Values**
w_{o}	0.5
w_{r}	0.1
w_{a}	1.0
w_{pc}	0.0
$w_{\mathrm{pc}}^{\mathrm{min}}$	-1.0
$w_{\mathrm{pc}}^{\mathrm{max}}$	1.0
$\theta_{\mathrm{post}}^{\mathrm{pc}}$	-1.0
w_{pa}	$0.9(0.3)$
$w_{\mathrm{pa}}^{\mathrm{min}}$	$0.1(0.05)$
$w_{\mathrm{pa}}^{\mathrm{max}}$	1.0
$\theta_{\mathrm{post}}^{\mathrm{pa}}$	$0.5(0.1)$
d	1.0
τ_r	20000.0
α	1.2
β	-0.55
σ_ξ	0.1
η_{critic}	$0.01(0.125)$
η_{actor}	$0.2(0.250)$
δt	$19.0(0.0)$
**E**	**Input: discretize adapter**
**Name**	**Values**
σ_x<br>σ_y	0.01 (0.2)<br>– (0.2)

Table S3. Table of the network parameters used for both tasks (according to Nordlie et al. (2009)), continued. Values in brackets are used for the *MountainCar* environment.

	OpenAI Gym
**Name**	**Values**
Version	0.8.1
	**MountainCar**
**Name**	**Values**
Version	0
Max episode steps	None
Initial reward*	-1.0
Final reward*	-0.4
Inter-trial duration*	0.4
Update interval (env runner)*	0.02
	**FrozenLake**
**Name**	**Values**
Version	0
Max episode steps	None
Slippery	False
Final reward null*	-0.1
Inter-trial duration*	0.1
Update interval (env runner)*	0.1

Table S4. Table of the environment parameters. Values marked with * indicate values for the ZeroMQ wrapper.
